# Knee function and quality of life in adolescent soccer players with Osgood Shlatter disease history: a preliminary study

**DOI:** 10.1038/s41598-023-46537-7

**Published:** 2023-11-06

**Authors:** Rafał Bruzda, Bartosz Wilczyński, Katarzyna Zorena

**Affiliations:** 1 Gdansk College of Health, 80-335 Gdansk, Poland; 2 Department of Immunobiology and Environment Microbiology, Medical University of Gdansk, 80-219 Gdansk, Poland

**Keywords:** Quality of life, Rehabilitation, Health care, Rehabilitation

## Abstract

The aim of the study was to assess psycho-physical deficits among young athletes with history of Osgood Shlatter Disease (OSD-H) compared to adolescents without history of knee pain (Control group). Eighteen participants were recruited from a group of 80 young (U14–U17) male soccer players. Medical records, and Knee Osteoarthritis Outcome Score (KOOS) were performed from both groups. The tests were conducted to assess the anthropometric characteristics, knee and ankle range of motion (Ely’s and Weight Bearing Lunge tests), and muscle power of the lower limbs (Leg Press, Single Leg Countermovement Jump, and Single Leg Hop for Distance). Results showed that overall patient-relevant outcomes following OSD (87%) were significantly lower than in the Control group (95%) (p < 0.05). Scores of the KOOS subscales revealed that Quality of Life and Function, Sports and recreational activities were significantly lower in the OSD-H group (p < 0.05). Although, there were no differences in subscale scores for Pain, Symptoms and Daily Activities. There were, also no difference between groups in range of motion angles and muscle power of the lower limbs values. The preliminary results support existing knowledge indicate that OSD may have a long-term influence on function, sports and recreational participation, and quality of life on young athletes.

## Introduction

The popularity of soccer among children and adolescents is steadily increasingworldwide^1^. However, concerns arise from sport specialization, heightened training loads, external pressures (from coaches and parents, and various biomedical factors, which can potentially impede the holistic development of young athletes. Our research team^2–5^ along with numerous other researchers^1,6–9^ have delved into the adverse ramifications of intense soccer practice. The main concerns appear to include sports burnout and musculoskeletal injuries. Notably, a significant number of young athletes terminate their sporting endeavors around the age of 15, with injuries peaking around 13 years of age among soccer players^10^.

Osgood-Schlatter disease (OSD) stands as one of the most prevalent non-traumatic knee injury among highly active children and adolescents^7^. Despite its recognition for over a century, the precise etiology of OSD remains elusive. Currently, the theory most widely accepted by researchers suggests that prolonged and/or repetitive stress on the patellar tendon may contribute to OSD. The reason could be strong and forceful pulls (traction forces) of the quadriceps muscle and patellar tendon on the apophysis in the anterior part of the tibial tuberosity^11^. Additionally, the natural progression of changes during puberty may also influence the onset of OSD^12^.

The duration of symptoms during OSD varies across studies. The minimum duration of pain has been reported to be about 6 weeks, but it can last for more than 6 months. There have been reported cases in which pain lasted much longer—a year or even 2 years^8^. Even after treatment intervention, symptoms may remain in some individuals. Rathleff et al. a showed positive effect of decreased symptoms in 80% of subjects after 12 weeks and in 90% after 12 months after activity modification and knee strengthening intervention^13^. Conversely, Krause et al.^11^, showed that 90% of patients with OSD who were treated conservatively, experienced complete resolution of symptoms within approximately 1 year of symptom onset, although deficits in strength and knee function persisted for much longer.

Moreover, recent studies have established an association between OSD and diminished quality of life, reduced function, muscle strength, and restricted range of motion in the lower limb joints^9,13^. Findings from a 2021 meta-analysis by Holden et al. revealed that about 50% of adolescents with a history of non-traumatic anterior knee pain (OSD and patellofemoral pain) experience pain and function limitations 12 months after diagnosis^7^. An analysis by Djurtoft et al. also revealed that a group of adolescents undergoing OSD and Patellofemoral pain were associated with negative psychological impact and impaired Quality of Life^14^.

One of the potential factors associated with OSD described in the literature is a "shortened" quadriceps muscle^15^. De Lucena et al. showed that the extensibility of the quadriceps muscle was significantly lower in the OSD group than in the control group^9^. Additionally, Enomoto et al.^16^ suggested that increased stiffness of the rectus femoris muscle under stretching conditions (at 45° and 90° flexion) may be linked with the development of OSD.

Another identified deficit that in individuals with OSD is limited dorsiflexion at the ankle^17^. This may involve a compensatory mechanism that increases the traction load on the tibial tuberosity. Deficits in ankle dorsiflexion lead to increased knee flexion, inversion (external rotation) of the tibia, and pronation of the foot during running. As a result of these actions, there may be increased traction on the quadriceps muscle attachment to the tibial tuberosity^15,18^. A restriction of dorsiflexion of 10° or more may predispose to anterior knee pain^19^.

Risk factors for OSD may ecncompass weight, height, and BMI. In a study by Omodaka et al., athletes with symptomatic OSD showed significant associations with higher body weight, height, and their BMI exceeding that of the control group (without a history of OSD)^15^. The experience of suffering OSD may be associated with prolonged reduced knee and hip joint strength over time. Recent studies have shown that adolescents with anterior knee pain had reduced lower limb strength compared to the controls^6^.

The objective of this study was to assess psycho-physical deficits in young athletes with a history of OSD in comparison to adolescents without a history of knee pain. Our primary aim was to examine differences in the scores on short and long-term patient-relevant outcomes in the Knee Injury and Osteoarthritis Outcome Score (KOOS) questionnaire (perceived functional deficits, quality of life, symptoms, pain)^20^. In addition, the study aimed to assess the muscle power of the lower limbs, the range of motion of the ankle and knee joints, and the flexibility of the quadriceps muscle. Implicitly, the asymmetry between the dominant and non-dominant lower limb in terms of the above variables was also assessed.

The results of this preliminary study will allow an attempt to understand the limitations of OSD in adolescents. More importantly, the results will allow the design of a randomised controlled trial evaluating conservative interventions for OSD.

## Methods

### Subjects

A control group of soccer players without a history of anterior knee pain was also included, comprising nine young players. This control group was drawn from the same team and age group as the OSD-H group. All participants (n = 18) had a median age of 16 (range: 14–17) and a BMI of 21 (range 18–23). Each player engaged in three weekly training sessions during the competitive season, with one match per week on an artificial playing surface. The athletes were assigned to one of the groups: OSD-H (with a History of Osgood Shlatter Disease) or control (players who had never had OSD, anterior knee pain, or similar specific symptoms in the past).

Inclusion criteria for the study: clinically and imaging confirmed OSD (for OSD-H group only), informed and written consent from the guardian/parent for the child's participation in the scientific study, a recent positive examination by a sports medicine physician, the absence of any pain and/or injury during the course of the study, and no history of lower extremity or trunk surgery within the past 2 years.

### Design and setting

A cross-sectional study was conducted involving two groups: OSD-H and Control, each comprising nine young athletes. The athletes were assessed during their training and competition days off at the Rehabilitation and Training Centre in the winter of 2022/23. This study was a component of a scientific project approved by the Bioethics Committee for Scientific Research at the Medical University of Gdansk (approval number: NKBBN/241/2023). Throughout the study, adherence to the Helsinki guidelines was maintained. Informed consent statements were obtained from all participants and their legal guardian(s). Prior to commencing the study, the participants were given a detailed explanation of the study and its aims. Medical records of participants with a history of OSD (including MRI, ultrasound, or X-ray) were collected by R.B. The interview and survey data were collected first. The survey contained data on the player's leading leg, injuries that occurred in the lower extremities and trunk within the past 2 years, pain experienced on the day of the study, and weekly participation in sporting activities (divided into soccer training, physical education classes, strength and conditioning training, training for other sports, and matches). Additionally, the survey inquired about rehabilitation received after the OSD diagnosis.

In addition, the questionnaire was expanded to include questions about the history of OSD, which included information about the course and duration of the condition, the cessation of the training, the exact time it occurred, any rehabilitation received, and the number of hours per week spent in sport prior to the onset of the condition. The athletes were then asked to complete the Knee Injury and Osteoarthritis Outcome Score (KOOS) questionnaire. All questionnaires and scales were administered face-to-face.

In the next step, the athletes underwent body composition analysis (InBody 270 analyzer, InBody Co., Seoul, Korea). This included measurements of height, body weight, skeletal muscle, BMI, and body fat percentage. On the basis of the data collected on the day of the measurement (standing height, sitting height, body weight and chronological age), the biological status was calculated according to the formula of Mirwald et al.^21^. The procedure forecasts the maturity shift (before or after of reaching peak height velocity (PHV), for each participant)^2^. The final stage was physical testing, i.e. range of motion of the knee and ankle joint and measurement of lower limb muscle strength in the single-leg tests. Figures illustrating all the tests are included in the supplementary material S1.

### KOOS scale

Participants completed the KOOS questionnaire^20,22^. The KOOS contains five subscales that assess: (1) pain, (2) symptoms, (3) daily activities, (4) sports and recreational activities, and (5) quality of life. The respondent's well-being during the day of the survey is also assessed. The survey form provided the opportunity to give answers ranging from 0 to 4 points (5-point Likert scale)^20^. The subscale scores were calculated as the sum of the included items according to the KOOS user manual. These scores were then converted to a scale of 0–100 (0 representing very severe knee problems and 100 representing absence of knee problems)^22^. Data from the systematic review and meta-analysis showed that all subscales of the KOOS had high test-retest reliability (ICC range 0.85–0.9)^23^.

### Ely’s test

Ely’s test assesses the flexibility of the quadriceps muscle and the range of flexion of the knee. The range of motion was measured with an 18-inch flexible and adjustable goniometer. The procedure for performing the test was as follows: the examiner placed 1.5x1.5 cm pieces of self-adhesive Tape on the head of the fibula, the greater trochanter of the femur, and the lateral ankle as landmarks. The participant was then instructed to maintain neutral pelvic alignment and anterior hip joint contact with the rehabilitation couch while actively performing maximum knee joint flexion. The degree of flexion was measured around a pivot axis running through the head of the fibula, while the two arms of the measuring equipment were positioned toward established landmarks: the greater trochanter of the femur and the lateral ankle. The test was performed bilaterally^24^. Ely’s test showed moderate levels of inter and intrarater reliability (ICC = 0.66–0.69) in the study of Peeler and Anderson 2008^24^.

### WBLT

The Weight Bearing Lunge Test (WBLT) assesses ankle dorsiflexion range of motion. The test involves touching the wall with the front of the flexed knee joint in a lunge position from the farthest possible position of the foot relative to the wall. The condition is that the heel of the test leg must not move away from the ground during the test while the foot is on the designated line perpendicular to the wall. The maximum distance of the toe from the wall was measured with a yardstick. Results were expressed in centimeters. Each participant performed 3 trials (the average of the results was analyzed). The weight-bearing lunge test has high inter-clinical reliability (ICC = 0.80–0.99) and good intra-clinical reliability (ICC = 0.65–0.99)^25^.

### Leg press—peak power output

The subjects' peak power output of the lower limbs was assessed with a Keiser Air420 air crane (Keiser Corporation, Fresno, CA, USA). The Keiser Leg Press crane consists of two independent plates allowing the two limbs to be evaluated separately in terms of strength, power, and asymmetry between the lower limbs. The load of each attempt was set at 120% of the subject's body weight. The Keiser pneumatic resistance system allows for precise load determination in 1 kg increments and displays peak power output in watts (W).

Prior to the actual tests, each participant performed one repetition with a load of 30/60/90% of body weight with the right and left leg in succession (test preparation). The correct starting position for the test was considered to be sitting with the knee joints flexed to 90°, while the final position was considered to be when the knee joints reached full extension. Each participant was informed that the thrust movement (i.e. the concentric phase) was responsible for the peak power value and that they should perform it with maximum intention. Between each actual trial, the participants had a 3-minute break. They completed a total of 3 trials. The best result from the three trials was used for analysis. The good reliability (ICC = > 0.76) and repetitiveness of the device have been confirmed in previous studies^26,27^.

### Single leg countermovement jump

The countermovement vertical jump (CMJ) was used to test the single leg power of the lower limbs. The subjects were asked to position themselves on a contact mat (Fusion Sport Smart Jump mat, Fusion Sport, 2 Henley ST, Coopers Plains, QLD, 4108, Australia) with feet positioned at hip width.

Participants were then told to keep their hands on their hips during the jump-off, flight phase and landing. A trial was considered passed if the participant kept his or her knee joints straight during flight and the initial phase of the foot with the contact mat during landing. There was a 2-minute break between each trial. The highest jump values (cm) from each participant's 3 trials were submitted for analysis^5^. The countermovement jump allows for a reliable assessment of vertical jump height calculated from flight time^28,29^. Reliability examined in the previous study was determined to be high (alfa coefficient between 0.93 and 0.96)^28^.

### Single leg hop for distance

Prior to the single-leg distance jump test, study participants were asked to stand at a designated starting line in a single-leg standing position (the supporting limb being tested) with their hands placed on their hips. Subsequently, the participants were told that their task would be to perform the longest possible forward jump along with landing on the same limb. The condition to pass the repetition was to maintain a stable position for 2 s after landing^2^. Participants performed three attempts on each limb (alternating, to reduce fatigue). There was a 1-minute break between each attempt. The length of each jump was measured in centimeters using a measuring tape. The longest distance counted in centimeters for each limb was submitted for analysis. The single-leg distance jump test has demonstrated good to excellent test-retest reliability (ICC = 0.84–0.98) in previous studies^30^.

### Statistical analysis

All variables were tested for normality of the data (Shapiro-Wilk test, assessment of symmetry, skewness and kurtosis of the distribution). Variables that were parametric were described as mean and standard deviation.Variables that were non-parametric were reported as medians and interquartile ranges. Due to the small sample size, copmparative statistics for non parametric variables were used. The Mann-Whitney test was used for non-parametric variables between group comparisons. Statistical analysis was performed in Statistica (Statistica 13, StatSoft) software. The threshold p-value was set at p < 0.05.

## Results

### Participant characteristics

Of the eighty young male soccer players (U14–U17) from Arka Gdynia SI, nine (11.2%) had a history of OSD and were included in the study. The characteristics of the participants were presented in Table 1. There were no statistically significant differences between the groups in anthropometric values and sports/soccer details (p > 0.05).

**Table 1:** Descriptive data of participants.

Variables (median (IQR))	OSD-H (n = 9)	Control (n = 9)	p*
Age (years)	16.0 (1.0)	16.0 (1.0)	0.724
Age PHV (years)	14.5 (0.8)	14.2 (0.6)	0.596
Maturity offset (years)	1.4 (1.5)	1.2 (0.85)	0.929
Height (cm)	175.0 (10.0)	175.0 (7.0)	0.691
Body mass (kg)	65.0 (3.4)	66.0 (15.4)	0.825
BMI	21.3 (2.3)	20.0 (3.4)	0.401
Body fat percent (%)	11.0 (2.9)	10.5 (0.6)	0.596
Muscle mass (kg)	33.5 (2.5)	31.8 (6.9)	0.536
Dominant lower limb, right (%) (n)	9 (100%)	6 (66.6%)	0.058
Soccer experience (years)	10.0 (0.0)	10.0 (1.0)	0.508
Time dedicated on soccer per week (h)	10.0 (0.0)	10.0 (0.0)	0.724
Time dedicated to sports (all disciplines) per week (h)	12.0 (2.0)	10.0 (2.0)	0.724

Data are median and interquartile (IQR).

*Age PHV* age peak height velocity, *BMI* body mass index.

*Mann–Whitney U test for nonparametric variables.

### OSD-H group—detailed retrospective information

Additional details from the participants' self-report questionnaire are shown in Table 2. Among the 9 players in the OSD-H group, 5 (55.6%) of them underwent the OSD (range: 1–4 years ago) at age 13, 2 (22.2%) at age 12, and 22.2% (n = 2) at age 14. Respondents indicated that their break from training due to OSD most often lasted 3 months (n = 4), followed by 4 months (n = 2) and 6 months (n = 1), 17 months (n = 1) and 18 months (n = 1), respectively. To the question of whether pain recurred after undergoing rehabilitation and returning to training after undergoing OSD, only one athlete answered “no” (11.1%), while 8 athletes (88.9%) answered “yes”. All of the respondents indicated that the pain was present bilaterally, but in 77% of them (n = 7) it was stronger in the non-dominant limb, and in 23% (n = 2) of them it was stronger in the dominant limb.

**Table 2:** Detailed information about OSD-H group from questionnaire and interview.

Variables from the survey—retrospective variables	OSD-H group (n = 9)
What age were you when you underwent OSD?	13.0
How long was the break from training due to OSD? (months)	4.0 (min 3.0 max 18.0)
How many hours a week did you spend in soccer training before OSD occurred?	8.0
How many PE lessons did you have before the onset of OSD?	6.0
Did the pain complaints occur bilaterally?	8 (88.8%)
In which lower limb were the symptoms worse?	Left 6 (66.6%)
Was there rehabilitation after the OSD diagnosis?	9 (100%)

### Differences in the KOOS scale between groups

**Figure 1:**
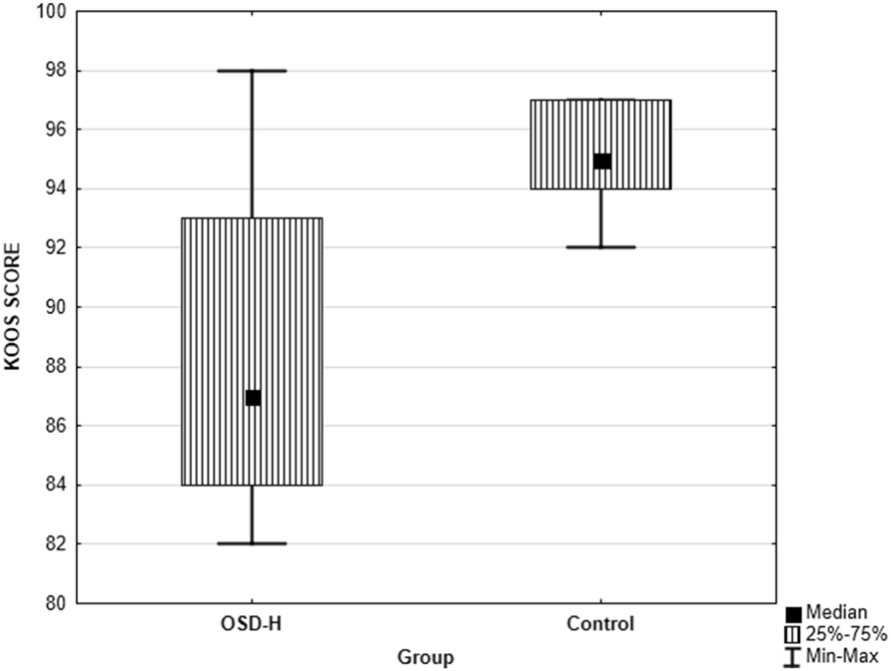
Differences in the KOSS Overall score between OSD-H and Control group.

The survey results were non-parametric, so they were presented as median and interquartile range (IQR) (Table 3). Data visualizations of the KOOS (Overall Score) results were presented using a box-and-whisker plot (Fig. 1).

**Table 3:** Patient-relevant outcomes following OSD–KOOS scale.

KOOS Median (IQR)	OSD-H (n = 9)		Control (n = 9)		Differences between groups
	(%)	points	(%)	points	p
Symptoms + Stiffness subtotal	89 (7)	34 (2)	93 (4)	35 (1)	0.310
Pain subtotal	94 (5)	17 (2)	97 (3)	18 (1)	0.093
Function, daily living subtotal	97 (1)	66 (1)	99 (3)	67 (2)	0.058
Function, sports and recreational activities subtotal	85 (10)	17 (2)	95 (5)	19 (1)	0.005*
Quality of life subtotal	75 (13)	13 (2)	94 (6)	15 (1)	0.030*
KOOS overall	87 (9)	145 (8)	95 (3)	154 (3)	0.010*

Mann–Whitney U test for nonparametric variables between groups. *p < 0.05.

A KOOS (Overall) scores between the OSD-H group (87, IQR: 9) and the Control group (95, IQR: 3) showed statistically significant differences (p = 0.010). Statistically significant differences were also found for the KOOS subscales. The subscale of the Function, sports and recreational activities shown difference between the OSD-H group (85, IQR: 10) and the control group (95, IQR: 5) (p = 0.005). On the KOOS Quality of Life Scale subscale, there was the difference between the OSD-H group (75, IQR: 13), and the control group (94, IQR: 6) (p = 0.030).

There was no difference in scores for the KOOS Pain and Symptoms Scale Subscales between OSD-H and the control group (p = 0.093 for Pain, and p = 0.310 for Symptoms). Also, on the KOOS Symptom Scale subscale, there was no significant differences, however the p-value was close to the threshold value (p = 0.058).

### Differences between and within groups in the Physical abilities

There were no statistically significant differences between groups (p > 0.05) and between left and right limbs (p > 0.05) in the assessed variables of range of motion (Ely's test and WBLT) and lower limb muscle power values (Leg Press, SLHD, SLCMJ). Table 4 shows the means, standard deviations and exact p-value results.

**Table 4:** Lower limb function evaluation of the two groups.

Variables (median (IQR))	OSD-H (n = 9)	Control (n = 9)	p between groups	p between variables
Ely’s test, L (°)	121.0 (6.0)	120.0 (7.0)	0.270	0.349
Ely’s test, R (°)	125.0 (3.0)	118.0 (10.0)	0.200	
WBLT, L (cm)	10.0 (4.5)	10.0 (3.0)	0.724	0.690
WBLT, R (cm)	11.00 (2.5)	10.0 (0.0)	0.452	
Leg Press, L (W)	958.0 (285.0)	932.0 (213.0)	0.427	0.163
Leg Press, R (W)	948.0 (320.0)	898.0 (175.0)	0.480	
SLHD, L (cm)	180.0 (7.0)	173.0 (23.0)	0.289	0.695
SLHD, P (cm)	183.0 (12.0)	168.0 (17.0)	0.085	
SLCMJ, L (cm)	22.5 (2.4)	23.0 (2.1)	0.860	0.542
SLCMJ, P (cm)	23.6 (2.5)	24.0 (1.1)	0.791	

*WBLT* weight bearing lunge test, *SLHD* single leg hop for distance, *SLCMJ* single leg counter movement jump, *W* watts.

Mann–Whitney U Test and Wilocoxon test for non parametrics variables. *p < 0.05.

## Discussion

Based on our preliminary findings, the results indicated that overall patient-relevant outcomes following OSD (87%) were significantly lower than in the Control group (without a history of OSD) (95%). Considering the KOOS scale scores in detail, the Quality of Life and Function, Sports and Recreational Activities subscales were also significantly lower in the OSD-H group. Although, there were no differences in subscale scores for Pain, Symptoms, and Daily Activities (it is worth noting the closeness of the threshold p-value, p = 0.058). However, studies have shown that a cut-off of 8 points represents a clinically significant difference^22,31^. Our results showed lower values of differences between groups. Therefore, only the KOOS overall result (9 points of differences between OSD-H and Control group) can be considered clinically significant with caution. These findings may support existing knowledge and highlight to some extent the problem of the prolonged negative impact of OSD on adolescents.

Similar conclusions were drawn by Krause et al.^11^ who demonstrated that despite the fact that in 90% of the subjects the pain resolved after about a year after the onset of symptoms, these individuals still complain about knee function. The results of the Guldhammer et al. study also showed lower reported KOOS scale values (Sports/Recreation and QoL subscale) in patients with a history of OSD at 4-year follow-up^32^. The subjects in the Guldhammer et al. follow-up study who were pain-free showed an average KOOS score as 85 points^32^. These score were similar to our results (KOOS overall = 87 points)^32^. The data described in the study by Djurtoft et al. in which they examined the total score of KOOS QoL in 323 adolescents with OSD and patellofemoral pain, the result was a 51%^14^. We believe that results of participants of our study are still considered "good," however, statistically different from the group with no history of knee injury.

Kaneuchi et al.^33^ reported that the average age of boys and girls participating in sports in which OSD occurs was 12 years (incidence rate of 13.8% in boys and 11.4% in girls). Sports emphasizing activities such as jumping, kicking, changing direction, and running predominately have been associated with an increased risk of OSD^15,34,35^. Notably, soccer stands out as the sport in which OSD is most commonly described in the literature, accounting for 13.6% of all knee injuries in 12–15 year old players^36^. Moreover, bilateral OSD occurs in 20–30% of cases^9^. Our interviews revealed that athletes experienced OSD approximately at 13 years of age and had a break from sports activity from 3 to as long as 18 months. The pain was mainly present bilaterally, however, it was worse in the non-dominant limb. Guldhammer et al. study also found a similar average age of participants with OSD symptom onset at 12.6 ± 3.2 years, but, participants' knee symptoms were 51% unilateral and 49% bilateral. The reason for the occurrence of more severe pain in the non-dominant (supporting) lower limb in soccer may be indicated by the higher impact of traction forces caused by eccentric contraction of the quadriceps through the patellar tendon on the fully immature apophysis during the action of kicking the ball^37^.

Despite, recent evidence regarding the impact of OSD on the reduction of lower limb muscle power and/or strength^13^, in our study, adolescent with and without a history of OSD demonstrated non significant results in the Leg Press, SLHD, and SLCMJ scores. This happened despite the use of three different tests (leg press, horizontal, and vertical jump) to assess lower limbs power. The tests selected for evaluating lower limb power comprehensively address the study's objectives. The Leg Press—Peak Power Output test offers insights into overall lower limb power production, whereas the Single Leg Countermovement Jump and Single Leg Hop for Distance tests focus on unilateral power in dynamic scenarios. These tests together provide a multifaceted perspective on lower limb power, capturing a range of relevant movement patterns and intensities. The robustness and comparability of our results within the broader scientific context were ensured by their established validity in previous research^26–28,30^.

Moreover, the same situation was with knee and ankle range of motion, and quadriceps muscle flexibility results. The selected range of motion tests were chosen judiciously to comprehensively assess relevant joint mobility. The Weight Bearing Lunge Test (WBLT) specifically evaluated ankle dorsiflexion range of motion, a crucial parameter for various weight-bearing activities. Additionally, Ely's Test was employed to gauge knee range of motion, providing insights into the flexibility and functional capacity of the knee joint. These tests collectively offered a comprehensive evaluation of joint mobility pertinent to our study population. Their established validity in prior research ensures the reliability and comparability of our findings within the broader scientific context^24,25^. There were no significant differences in the WBLT between the OSD-H and the Control group. The absence of deficits (< 10 cm) in the range of dorsiflexion can be considered a positive aspect in both groups, due to the fact that the aforementioned limitation was considered a risk factor for anterior knee pain^38^.

The quadriceps muscle functions eccentrically while running until the propulsion phase commences at the point where the knee reaches its maximum level of flexion. Therefore, “shortened” quadriceps muscle may play a role in the development of the OSD. Knee ROM and quadriceps flexibility have been evaluated using Ely's test. Conclusively, both groups demonstrated comparable quadriceps flexibility results in our study. These findings are inconsistent with the study of De Lucena et al.^9^ in which a total of 956 participants aged 12–15 years were studied, where 93% of those with OSD presented with a decrease in quadriceps flexibility. The difference in the outcomes compared to the mentioned study may be due to a different test (adapted Thomas test versus Ely’s test) that evaluates quadriceps flexibility. The range of motion and power of the lower limb variables may have been influenced by the fact that all OSD-H group players reported undergoing rehabilitation in the information questionnaire. Conservative treatment included, among other things, elements of manual therapy and exercises which included work to improve range of motion and lower limb strength.

Our study indirectly sought to evaluate asymmetry in the assessed variables of lower limb range of motion and power. Interestingly, the results revealed no significant differences between the groups. This aligns the observed bilateral incidence of OSD but does not entirely correlate with the higher incidence of pain in the non-dominant (supporting) lower limb within the OSD-H group. It is worth noting that this was a preliminary study and therefore has certain limitations. Firstly, the conclusions drawn from the results cannot be extrapolated to practical implications. The assessors were not blinded, which increases the risk of bias. Additionally, as the participants were solely soccer players, it is not possible to make a comprehensive comparison with the results of studies including athletes from various sports. In our study, participants trained and played matches on an artificial surface. It is necessary to objectively evaluate the difference between the playing surfaces. Additionally, future studies must incorporate biomechanical analysis of the lower extremities to further understand the implications of playing on artificial surfaces. The results obtained from this study will be useful for large-scale cross-sectional and randomized trials.

## Conclusion

Preliminary findings indicate that 13-year-old football players commonly develop OSD, which affects both limbs but causes more severe pain in the non-dominant one. Athletes who previously experienced OSD (1–4 years ago) reported deficiencies in function, sports and recreational activities, and quality of life, while not exhibiting symptoms or pain on the KOOS scale. There were no significant between-group differences in the muscle power of the lower limbs and knee and ankle range of motion angles. Our findings may corroborate previous research indicating that OSD history could adversely affect the function, sports engagement, and quality of life of teenagers in the long term. The preliminary results of this study will allow for a future randomized controlled trial to investigate the impact of conservative treatment interventions for OSD.

### Supplementary Information

Supplementary Figures.

## Data Availability

The datasets used during the study are available from the corresponding author upon reasonable request. Additional materials have been included in the supplementary material S1.
