# Supplementary material for: Knee function and quality of life in adolescent soccer players with Osgood Shlatter disease history: a preliminary study
Source: Sci Rep. 2023 Nov 6;13:19200. doi: 10.1038/s41598-023-46537-7 (PMC10628204; doi:10.1038/s41598-023-46537-7)
Supplement: Supplementary file 1 — Supplementary Figures. [file 41598_2023_46537_MOESM1_ESM.pdf]

SUPPLEMENTARY MATERIAL

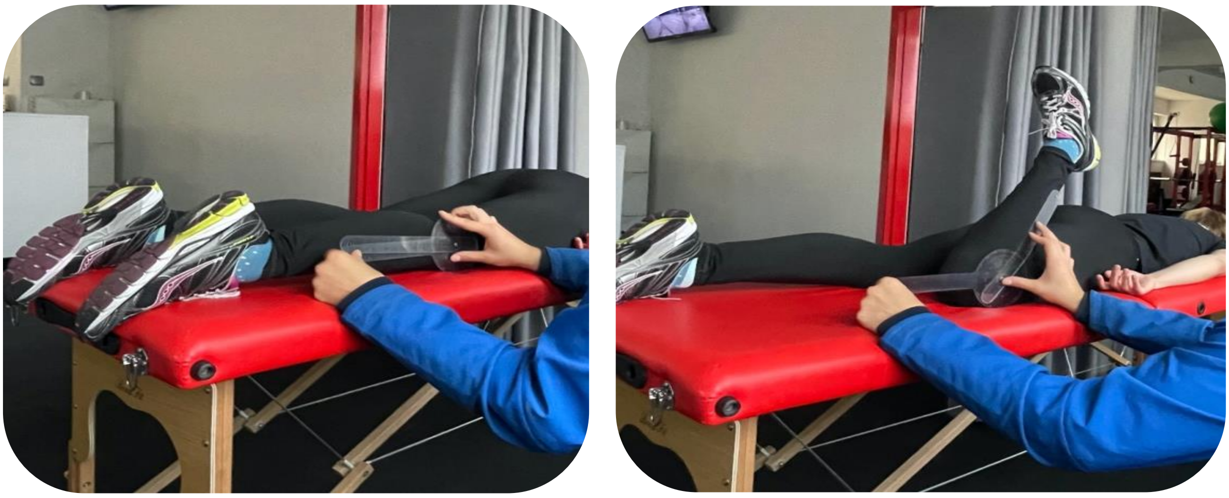

Supplementary Figure 1. Ely's test. Start (knee full extension) and end position (knee maximally flexed) of the test.

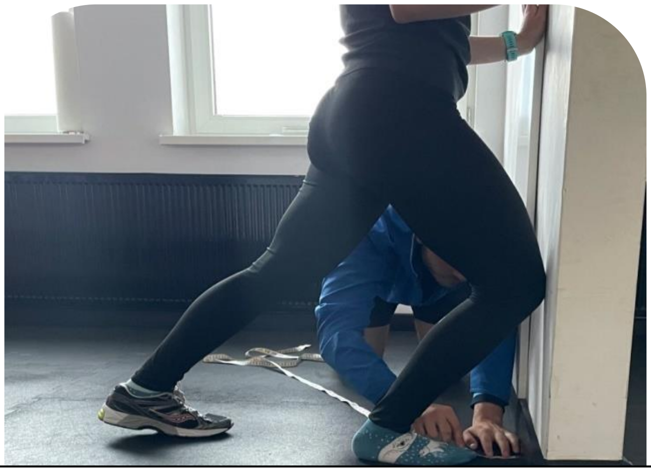

Supplementary Figure 2. WBLT test. End position (furthest point of the foot from the wall) of the test.

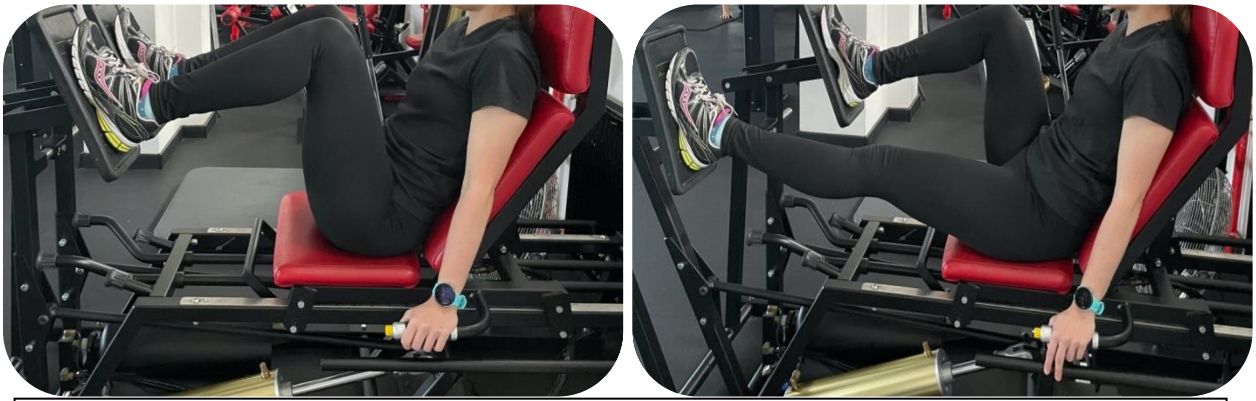

Supplementary Figure 3. Leg Press test (Keiser Air420 air crane). Start and end position (left knee extension) of the test.

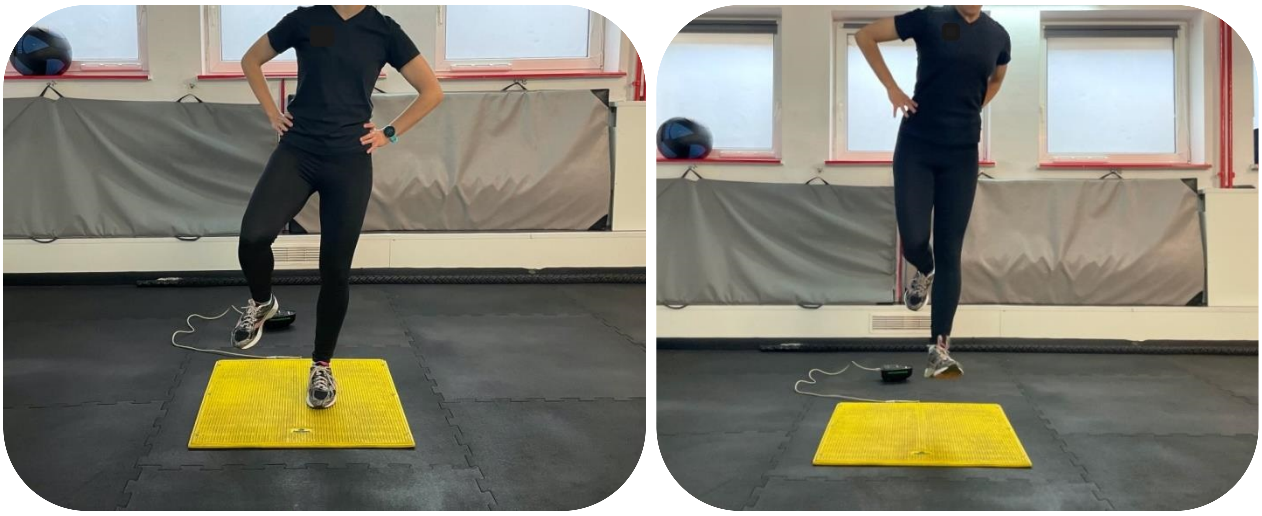

Supplementary Figure 4. Single Leg Countermovement Jump. Start and mid position (jumping off moment) of the test.

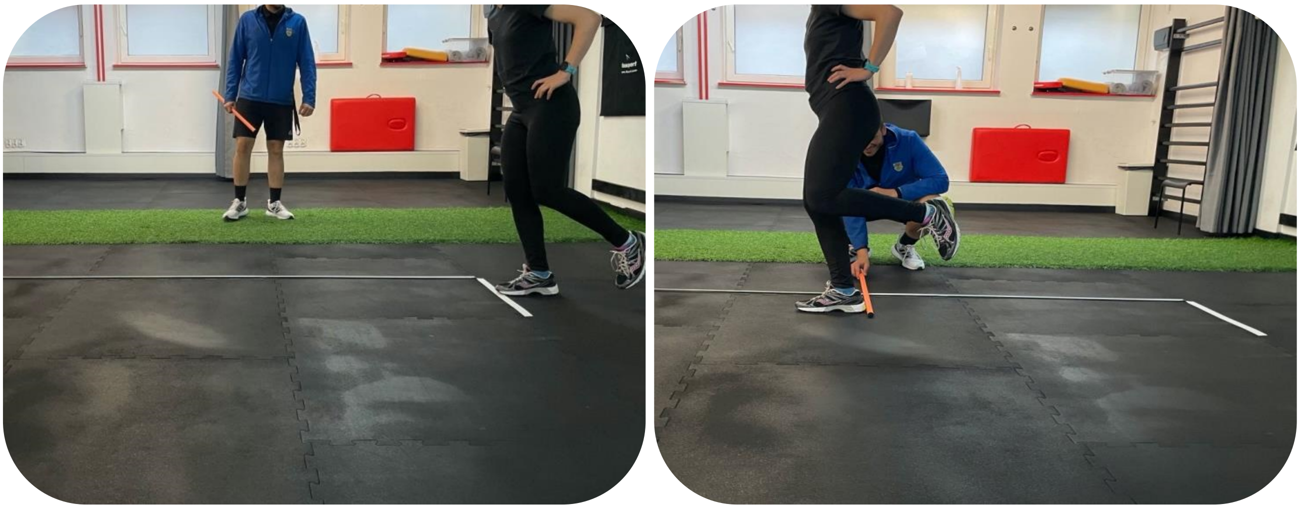

Supplementary Figure 5. Single Leg Hop for Distance. Start and end position of the test.
